# Audience attention in classroom peer presentations as a situated, role-dependent, and evaluative process

**DOI:** 10.3389/fpsyg.2026.1781721

**Published:** 2026-06-24

**Authors:** Jianxin Yang, Pingyan Li

**Affiliations:** 1 College of Foreign Languages, Zhangye, China; 2 School of Foreign Languages, Lanzhou University of Technology, Lanzhou, China

**Keywords:** attentional regulation, audience attention, grounded theory-informed qualitative research, peer presentations, perceived attention, student engagement

## Abstract

**Background:**

Peer presentations are widely used to promote active learning, oral communication, and peer interaction. Existing research has mainly examined presenters' performance, learning outcomes, and anxiety, while less attention has been paid to how non-presenting peers experience and regulate attention. Drawing on classroom attention, mind wandering, self-regulation, and student engagement research, this study examines audience attention as a situated and role-dependent process in peer-led instruction.

**Methods:**

This study adopted a grounded theory-informed qualitative design to develop a context-sensitive process model of audience attention during classroom peer presentations. Data included classroom observations, 16 semi-structured interviews, and 72 written narratives collected shortly after presentation sessions. Rather than treating these accounts as direct measurements of real-time attention, the analysis examined participants' perceived, recalled, and reconstructed attentional experiences through iterative coding, constant comparison, and triangulation.

**Results:**

Participants described audience attention as a dynamic, evaluative, and recursive process shaped by the social and epistemic conditions of peer-led instruction. Participants moved among focused engagement, intermittent attention, surface monitoring, and disengagement. Attentional investment was shaped by topic relevance, delivery accessibility, instructional legitimacy, internal vulnerability, and accountability cues. Disruption prompted minimal-cost self-regulation, externally triggered re-engagement, or disengagement when perceived instructional value was low. Evaluative outcomes were retrospectively linked to later expectations across presentation episodes.

**Conclusion:**

The proposed model extends attention and engagement research by showing how general attentional mechanisms are reorganized in peer-led instructional contexts, where knowledge authority is provisional, accountability is comparatively weak, and audience members act as learners, peers, and evaluators. The findings highlight the need to consider audience experience, presentation quality, and instructional design when implementing peer presentations.

## 1 Introduction

Peer presentations are widely adopted in classroom instruction across disciplines as a pedagogical strategy for promoting active learning, oral communication skills, and peer interaction. By positioning students as knowledge presenters, peer presentations are often assumed to enhance engagement not only for presenters but also for their classmates as audience members. Empirical classroom research has shown that peer presentations constitute an established instructional practice, particularly in postsecondary education, where they support students' academic communication, participation, and discourse socialization (Zappa-Hollman, 2007). However, while a substantial body of research has examined presenters' performance, learning outcomes, speaking anxiety, and feedback processes, comparatively less attention has been paid to how non-presenting peers experience, sustain, lose, and regulate attention while occupying the audience role.

Attention is a foundational condition for learning in classroom contexts, supporting comprehension, retention, and conceptual understanding. Yet classroom attention is increasingly recognized as temporally unstable and context-sensitive, rather than as a fixed or uniformly sustained state. Empirical studies of lecture settings have demonstrated substantial fluctuation in students' attentional focus over time, including frequent episodes of mind wandering even among motivated learners (Risko et al., 2012; Zanesco et al., 2024). More recent research has further modeled attentional fluctuation as a developmental process unfolding within and across instructional episodes, showing that mind wandering and attentional lapses are shaped by situational features, learner characteristics, and instructional conditions (Wammes and Smilek, 2017; Yang et al., 2025). These findings suggest that attention in instructional contexts is better understood as a dynamic process shaped by changing cognitive, affective, and contextual demands.

Existing theories of attention, mind wandering, self-regulation, situational interest, and student engagement explain how learners sustain, lose, and recover attention in educational settings. Engagement research conceptualizes classroom participation as behavioral, emotional, and cognitive involvement (Fredricks et al., 2004), while studies of interest and mind wandering highlight the roles of situational interest, fatigue, motivation, and internal readiness in attentional vulnerability (Hidi and Renninger, 2006; D'Mello, 2019). These perspectives establish attention as dynamic, regulated, and contextually embedded. However, they have less often examined how attentional processes are shaped by instructional role relations, especially in peer-led presentation contexts where students act as learners, peers, and evaluators.

Research on peer presentations has primarily emphasized presenters' speaking skills, content mastery, confidence, and academic communication competence. Studies of peer learning show that peer-led activities can promote higher-level cognitive processing when interaction is appropriately structured (King, 2002). Audience-oriented research has more often addressed perceived usefulness, peer feedback, or audience evaluation (e.g., Heideman and Laury, 2022), rather than the attentional processes through which listeners engage with peer-delivered content. Thus, the audience standpoint remains insufficiently understood, especially regarding how listeners interpret peer-delivered input, regulate attentional lapses, and form expectations toward later presentations.

Peer presentations provide a distinctive context for examining classroom attention because they temporarily reorganize instructional authority and accountability. Unlike teacher-fronted lectures, where institutional authority is stable and attention expectations are explicit, peer presentations place student presenters in a provisional instructional role. Audience members must navigate cognitive demands, peer-respect norms, variable presentation quality, and uneven perceptions of instructional legitimacy. Under these conditions, attention is unlikely to be explained solely by motivation, self-discipline, or general attentional capacity. Rather, it involves situated judgments about relevance, accessibility, authority, and the value of continued attentional investment.

Accordingly, the present study uses a grounded theory-informed qualitative approach to develop a situated process model of how audience members perceive, regulate, and evaluate attention in peer-led instruction. The study asks: (1) How do audience members perceive and regulate attention during classroom-based peer presentations? (2) What internal conditions, presentational features, and evaluative processes shape attentional fluctuation and engagement? (3) How are evaluations of peer presentations retrospectively linked to later attentional expectations? By foregrounding the audience role, this study shows how provisional instructional authority, weak accountability, variable delivery quality, and peer-relational norms reorganize attentional fluctuation, regulation, and evaluation in classroom peer presentations.

## 2 Literature review

### 2.1 Attention as a dynamic and temporal process in classroom learning

Attention has long been recognized as a foundational condition for learning, supporting comprehension, memory, and knowledge construction. However, contemporary research increasingly conceptualizes attention as a dynamic and temporally unfolding process that fluctuates in response to task demands and contextual conditions. Empirical studies conducted in classroom and learning settings have demonstrated that students' attention varies substantially within instructional episodes, with frequent shifts between on-task focus, partial engagement, and mind wandering (Risko et al., 2012; Trasmundi and Toro, 2023).

In educational contexts, mind wandering has been widely studied as a central manifestation of attentional fluctuation. It has been shown to emerge systematically over time and to be influenced by instructional design, learner interest, and cognitive load (Smallwood and Schooler, 2015). These findings challenge static views of attention and underscore the need to examine how attentional states develop, decline, and recover during classroom activities.

Building on this perspective, recent work has begun to model attentional trajectories across instructional time. For example, research on intra- and inter-lecture patterns of mind wandering in second language (L2) classrooms has shown that attentional lapses follow identifiable developmental trajectories shaped by situational features and learner characteristics (Yang et al., 2025). Such work highlights that attention is best understood as a process unfolding over time, rather than as an outcome measured at isolated points. However, most existing research continues to examine attention in teacher-led or individually framed learning contexts, leaving less understood how attentional dynamics operate when instructional authority is temporarily assigned to peers.

### 2.2 Interest, internal states, and attentional vulnerability

A substantial body of research has examined the internal conditions that shape attentional engagement, particularly learner interest, motivation, and fatigue. Interest has been shown to play a central role in sustaining attention, with both situational and individual interest reducing the likelihood of mind wandering and supporting deeper engagement (Hidi and Renninger, 2006; Perche et al., 2025). Related evidence also suggests that attentional failures, distraction, and mind wandering are closely connected to learners' ability to maintain cognitive involvement during learning activities (Ralph et al., 2019).

Beyond interest, internal states such as fatigue, emotional strain, and competing concerns have been identified as factors that lower attentional resilience. When cognitive or affective resources are depleted, learners become more susceptible to attentional breakdown, even in otherwise supportive instructional conditions (D'Mello, 2019). These internal factors rarely operate in isolation. Instead, they interact with situational demands, amplifying vulnerability to attentional fluctuation when instructional input becomes difficult to follow, monotonous, or insufficiently meaningful.

This line of research establishes that attentional engagement is conditional and context-sensitive, shaped by learners' internal readiness as well as by perceived demands and benefits of continued cognitive investment. These insights provide an important foundation for examining attentional processes in classroom activities that place sustained listening demands on learners, such as peer presentations. In such contexts, internal vulnerability may be intensified when peer-delivered input lacks clarity, relevance, or perceived instructional value.

### 2.3 Classroom attention and student engagement

Within educational psychology, attention is often discussed as a component of broader constructs of student engagement. Engagement has been conceptualized as a multidimensional phenomenon encompassing behavioral, emotional, and cognitive dimensions (Fredricks et al., 2004). From this perspective, attention is not merely a behavioral indicator, such as looking at the speaker, but also reflects cognitive investment, emotional involvement, and willingness to remain connected to instructional activity.

Research has increasingly distinguished between surface-level compliance and deeper forms of cognitive engagement. Students may display outward signs of attentiveness while engaging minimally with instructional content, particularly in contexts where social or normative expectations encourage visible participation (Skinner and Pitzer, 2012; Schnitzler et al., 2021). This distinction is especially relevant for classroom settings in which learners are expected to remain attentive over extended periods, even when instructional input varies in quality.

Despite these advances, much engagement research continues to rely on global or outcome-oriented measures, providing limited insight into how attention is experienced and regulated moment by moment. As a result, the micro-processes through which learners sustain, lose, recover, or strategically withdraw attention during specific instructional activities remain underexplored. This limitation is particularly important in peer-led instructional contexts, where visible participation may also reflect peer respect, classroom norms, or minimal compliance rather than sustained cognitive engagement.

### 2.4 Peer learning and peer presentations as instructional practices

Peer presentations are a common instructional practice in higher education and language classrooms, widely used to promote active learning, communication skills, and peer interaction. Research on peer learning has demonstrated that student-led instructional activities can support higher-level cognitive processing when appropriately structured (King, 2002). In language education, peer presentations have been shown to contribute to learners' academic discourse socialization and oral proficiency development, particularly in postsecondary contexts (Zappa-Hollman, 2007).

However, empirical research on peer presentations has predominantly focused on presenters rather than listeners. Studies have examined presenters' speaking proficiency, anxiety, feedback, and performance development, often framing peer presentations as opportunities for productive output and skill formation (Al-khresheh, 2024; Grieve et al., 2021; Heideman and Laury, 2022). While some research has addressed peer feedback or audience evaluation (van Ginkel et al., 2017), the attentional experience of non-presenting peers has received limited systematic attention.

This presenter-centered emphasis leaves a gap in understanding how peer presentations function as classroom learning events. Their effectiveness depends not only on presenters' performance but also on whether audience members attend to, interpret, and use peer-delivered content. Examining listeners' attentional processes is therefore essential for a fuller account of this instructional format.

Although classroom attention research has modeled attentional fluctuation and mind wandering, it typically conceptualizes learners as individual recipients of instruction and rarely differentiates instructional roles. Existing attention and engagement frameworks explain general mechanisms, including temporal fluctuation, internal vulnerability, regulatory effort, and surface vs. deep engagement (Fredricks et al., 2004; Smallwood and Schooler, 2015; Skinner and Pitzer, 2012; Yang et al., 2025). However, they offer less insight into how these mechanisms are reorganized in peer-led episodes, where instructional authority is provisional, presentation quality varies, and audience accountability is less explicit than in teacher-fronted instruction.

In peer presentation contexts, audience members are learners, peers, and informal evaluators simultaneously. Their attention may therefore involve not only cognitive regulation but also judgments of relevance, accessibility, legitimacy, and peer responsibility. The present study therefore develops a situated process model to examine how broader attentional processes are perceived, narrated, and regulated under the social, epistemic, and accountability conditions of peer-led instruction.

## 3 Method

### 3.1 Research design

This study adopted a grounded theory-informed qualitative design to explore how audience members perceived, reconstructed, and regulated attention during classroom-based peer presentations. Given the process-oriented and insufficiently specified nature of audience attention in peer-led instructional contexts, this approach was deemed appropriate for capturing learners' situated experiences, evaluative judgments, and regulatory strategies.

Rather than testing predefined hypotheses or applying an existing attention model deductively, the study aimed to develop a context-sensitive process model explaining how attentional fluctuation, regulation, and evaluation are organized in peer presentation contexts. Grounded theory served to elaborate, not replace, existing attention, mind-wandering, self-regulation, and engagement frameworks. These theories functioned as sensitizing concepts rather than fixed coding categories, allowing the analysis to remain open to participants' accounts while situating the model within established research. Multiple qualitative data sources were analyzed through iterative coding and constant comparison, allowing for triangulation and strengthening analytic robustness.

### 3.2 Context and participants

The study was conducted in undergraduate courses at a comprehensive university. Four course types were included: Comprehensive English, Literature, Introduction to Research Methods, and Linguistics. These courses were selected because they regularly incorporated peer presentations and represented language-focused, content-based, and skill-oriented learning contexts.

Course selection was initially purposive rather than purely theoretical. Courses were identified through consultation with instructors who regularly used peer presentations, ensuring that participants had repeated experience as audience members. During data collection, follow-up interview participants were selected in a theoretically informed manner, with attention to emerging categories such as visible attentional fluctuation, surface monitoring, re-engagement, and disengagement. Thus, the sampling process combined purposive course selection with theoretically informed selection of focal interview cases.

Each class included approximately 30–40 students. Peer presentations were individual presentations, usually lasting 3–5 min per presenter. Although each presentation was brief, several were often arranged within the same class session, creating repeated audience-listening demands. Presentations were delivered in English or Chinese, depending on course requirements, and presenters usually used PowerPoint slides.

The presentations contributed to regular course assessment, but accountability was stronger for presenters than for audience members. Audience members were expected to listen and could raise questions, join discussion, or offer brief comments after presentations. However, audience tasks were not always formalized through compulsory note-taking, written evaluation sheets, or individual assessment. Audience attention was therefore expected but not continuously monitored.

Teacher involvement was present but limited. Instructors usually assigned the task in the previous class, explained general requirements, and provided feedback after presentations. During presentations, teachers generally allowed students to complete their delivery, intervening mainly for clarification, time management, or classroom guidance. Device use was not strictly regulated: students were expected to avoid unrelated phone use, but discreet device use was not always explicitly policed. These conditions made peer presentations a useful context for examining audience attention under moderate instructional expectations, variable presentation quality, and relatively weak audience accountability.

Participants were undergraduate students from first to fourth year who had experience serving as audience members in the selected courses. Participation in interviews and written narratives was voluntary. Informed consent was obtained before individual-level data were collected, and participants were assured that their responses would remain confidential and would not affect course assessment.

### 3.3 Data sources and data collection

#### 3.3.1 Data sources

To capture audience attention from multiple perspectives, the study drew on semi-structured interviews and written narrative accounts within a classroom-based observational design. The dataset comprised 16 interviews and 72 written narratives, yielding approximately 38,942 words. These sources were combined to examine both visible engagement cues and students' retrospective accounts of attentional experience.

Classroom observation was conducted during peer-presentation sessions, focusing on visible fluctuations in engagement, such as posture change, gaze withdrawal, intermittent slide checking, device use, peer whispering, and shifts between monitoring and apparent disengagement. These behaviors were not treated as direct evidence of students' internal cognitive states. Rather, they provided contextual cues for identifying attentional episodes to be explored through interviews and narratives.

Students who exhibited salient attentional changes were purposefully selected for follow-up interviews. To avoid focusing only on disengagement, the interview sample also included students who appeared to maintain relatively stable attention. This enabled comparison across different attentional trajectories, including sustained engagement, intermittent attention, surface monitoring, re-engagement, and disengagement.

Semi-structured interviews elicited participants' perceived attentional experiences, regulatory responses, and evaluative judgments. Prompts invited them to reconstruct attentional shifts, perceived triggers, attempts to regulate or disengage attention, and evaluations of peer presentations. Other audience members produced written narratives after the same sessions, describing what they attended to, when attention shifted, how they responded, and whether the presentation was worth sustained attention.

Combining observation-informed interviews with written narratives enabled triangulation across observed engagement cues, retrospective elaboration, and self-reported cognitive experience. This design captured both salient individual cases and broader patterns of perceived audience attention.

#### 3.3.2 Timing of data collection and recall considerations

Both interviews and written narratives were collected immediately after class sessions in which peer presentations took place. This near-event elicitation strategy was adopted to reduce retrospective distortion and memory decay.

To minimize reactivity, students were not informed before the observed sessions that the study specifically focused on audience attention. They were informed more generally that the study concerned classroom learning experiences and peer-presentation participation. Interviews and narratives were elicited only after the sessions, when participants were invited to reconstruct naturally remembered moments, attentional shifts, and situational cues from the just-completed class.

Participants' accounts were treated as situated reconstructions of attentional experience rather than direct records of real-time cognitive states. This distinction was important because attentional fluctuation, especially when involving mind wandering or low-level monitoring, may not be fully accessible to conscious introspection. The analysis therefore focused on how students perceived, recalled, and made sense of attentional fluctuation and regulation. To reduce over-interpretation, interview and narrative data were compared with observation notes and recurring cross-case patterns. Interpretations were developed only when reported experiences, observed cues, and cross-case similarities converged.

Data were collected mainly in Chinese, although some course-related expressions and presentation content involved English. Quotations used in the manuscript were translated into English by the authors and checked against the original Chinese for semantic accuracy, with minor adjustments made only for readability. Participant quotations are identified by data source: P refers to interview participants and N refers to written narrative accounts. Numbers are anonymized source identifiers rather than chronological order of data collection.

### 3.4 Data analysis

Data analysis followed a grounded theory-informed procedure involving open, axial, and selective coding. Analysis proceeded iteratively alongside data collection, with constant comparison used to refine codes, compare cases, and examine relationships across interviews, written narratives, and classroom observation notes.

During open coding, interview transcripts and written narratives were read line by line to identify meaning units related to perceived attentional experience. Initial codes stayed close to participants' expressions and covered attentional states, visible engagement cues, influencing conditions, regulatory responses, and evaluative judgments. Observation notes were used to contextualize participants' accounts, not to infer internal cognitive states directly.

During axial coding, related codes were clustered into higher-level categories through a conditions-actions/interactions-outcomes logic. These categories included internal vulnerability, external and presentational conditions, attentional fluctuation, surface monitoring, minimal-cost regulation, externally triggered re-engagement, pragmatic disengagement, and evaluative outcomes. During selective coding, these categories were integrated around the core category of “the situated process of perceived audience attention in peer-presentation contexts,” which informed the final process model.

To make the coding process more transparent, Table 1 illustrates the analytic chain from data excerpts to initial codes, focused categories, and model components.

**Table 1:** Example coding chain from data excerpts to model components.

Data excerpt	Initial code	Focused category	Model component
“I would look up to see where they were talking about.”	Checking presentation progress	Surface monitoring	Attentional fluctuation
“If the PPT was full of text… I found it hard to keep up.”	Difficulty following dense slides	Delivery accessibility	Initial orientation/evaluative judgment
“When the teacher reminded us… attention was pulled back.”	Teacher-triggered refocusing	Accountability cue	Externally triggered re-engagement
“I let myself get distracted… and organized materials for my own presentation.”	Reallocating attention to another task	Pragmatic disengagement	Regulatory response

Coding was conducted by two researchers. Both independently coded a shared subset of interviews and written narratives to calibrate code definitions and identify ambiguous cases. Inter-coder agreement for this subset was acceptable (Cohen's kappa = 0.78). The remaining data were then coded and cross-checked iteratively. Disagreements were resolved through discussion, comparison with original excerpts and observation notes, and refinement of category boundaries. The kappa value supported coding consistency, while final category development relied on constant comparison and theoretical refinement. Saturation was judged when additional data no longer generated substantively new codes or relationships, and when the main process components recurred across courses and participant accounts.

### 3.5 Trustworthiness and ethical considerations

Several strategies were used to enhance trustworthiness and rigor. Constant comparison was applied across participants, data sources, and course contexts to avoid conclusions based on isolated cases. The analysis prioritized attentional process chains, including attentional change, regulation or disengagement, and evaluative outcomes. Analytic memos and the coding chain documented how raw excerpts were linked to codes, categories, and model components.

Triangulation across interviews, written narratives, and classroom observations strengthened credibility. Self-reports were treated as perceived and reconstructed accounts rather than direct measurements of real-time attention. Observation notes contextualized participants' accounts, while claims about attentional investment and evaluation were developed only when similar patterns appeared across participants and data sources.

Ethical approval was obtained in accordance with institutional guidelines, including approval of the limited disclosure used during classroom observation. To reduce reactivity, students were initially informed that the study concerned classroom learning experiences and peer-presentation participation, rather than audience attention specifically. Before interviews and written narratives were collected, participants were debriefed about the study focus, informed of their voluntary participation and right to withdraw, and assured that responses would remain confidential and would not affect course assessment.

## 4 Results

### 4.1 Overview of analytic findings

The analysis indicated that audience attention in this instructional format was experienced and narrated as a dynamic, evaluative, and recursive process. Classroom observations identified visible shifts in engagement, while interviews and written narratives clarified how participants interpreted these shifts. Across the data, attentional engagement was linked to three interrelated dimensions: (a) the perceived instructional legitimacy of peer-delivered content, (b) the accessibility of presentation delivery and materials, and (c) classroom accountability cues.

More specifically, participants described attentional engagement as shaped by the interaction of internal states, external presentational conditions, and retrospective judgments about whether continued attentional investment was worthwhile. For example, one participant recalled shifting from active listening to distraction after approximately 2 min, followed by watching short videos on a mobile phone, and regaining attention only during the question-and-answer phase:

“*After about two minutes, I took out my phone and watched short videos. It was hard to refocus until the presentation moved into the Q&A section*” (P07, interview).

This was interpreted as a reconstructed account of disengagement and re-engagement, rather than a direct measure of real-time attention.

Through iterative coding and constant comparison, the findings were organized around interrelated themes describing how audience attention was oriented, how it fluctuated over time, how disruption was regulated or allowed to continue, and how evaluative outcomes were retrospectively linked to later attentional expectations.

### 4.2 Attention as a dynamic and fluctuating process in the audience role

Participants reported moving across multiple attentional states within and across peer-presentation segments, including focused engagement, intermittent attention, surface monitoring, and disengagement. Focused engagement was associated with looking at slides, taking notes, or following the speaker's explanation. Reduced engagement involved partial monitoring, gaze withdrawal, device checking, or attention to unrelated tasks. These states were treated as reconstructed patterns of audience experience rather than direct measures of real-time attention.

#### 4.2.1 Surface monitoring as low-cost classroom presence

Disengagement during peer presentations was not always described as complete withdrawal. Even when sustained cognitive engagement weakened, participants often remained minimally connected by tracking slide transitions, speaker movement, or presentation stages. Analytically, this pattern was coded as surface monitoring: a low-cost form of classroom presence that maintained minimal alignment without sustained deep processing.

Several participants described losing focus while still checking presentation progress. One explained:

“*Each time it probably lasted about one minute, and then I would look up to see where they were talking about*” (P12, interview).

The same participant noted that re-entry was conditional:

“*If the presenter suddenly talked about something I was interested in, or if I reminded myself, I would return to the class*” (P12, interview).

Visual materials also served as anchors. Another stated:

“*After losing focus, I brought my attention back by looking at the PPT, looking at the PPT a few more times*” (N34, written narrative).

Participants distinguished visible presence from cognitive engagement. One stated,

“*It was hard for me to stay focused the entire time*” (N08, written narrative).

Another described partial engagement more explicitly:

“*I would occasionally look up at the PPT, but I didn't really pay attention to the content*” (N51, written narrative).

Such accounts suggest that looking at slides or tracking progress did not necessarily indicate deep processing.

In peer-presentation contexts, surface monitoring functioned as a pragmatic compromise. It allowed audience members to remain aligned with classroom norms and peer-respect expectations while reallocating cognitive resources when perceived instructional value declined. Rather than indicating inattentiveness alone, it represented low-cost attention management under variable presentation quality and limited audience accountability.

#### 4.2.2 Temporal attenuation and fragile re-engagement

Audience attention was frequently narrated as temporally unstable, with engagement weakening as presentation sequences unfolded. Several participants reported an initial phase of attention followed by gradual disengagement. One described this shift succinctly:

“*After a few minutes, I started to drift off* ” (N27, written narrative).

These accounts indicate that attentional decline was often progressive rather than abrupt.

Disengagement, however, was not described as irreversible. Participants reported brief episodes of re-engagement, though such recovery was often fragile and short-lived. As one explained,

“*If the presenter suddenly talked about something I was interested in, or if I reminded myself, I would return to the class*” (P12, interview).

Re-engagement was therefore usually prompted by renewed relevance, momentary salience, or self-initiated reminders.

These findings characterize audience attention in peer presentations as subject to temporal attenuation and conditional re-engagement. In the audience role, attentional recovery appeared possible, but it often depended on renewed relevance, stimulus salience, or self-reminding rather than sustained external accountability.

### 4.3 Evaluative orientations toward attentional investment in peer presentations

Participants' accounts indicate that attentional engagement in the audience role was closely tied to evaluations of peer-delivered input. Rather than describing attention as a purely automatic response, participants explained their engagement in relation to whether a presentation seemed relevant, accessible, and worth continued effort.

#### 4.3.1 Relevance and accessibility as dual evaluative dimensions

Two recurring evaluative dimensions shaped attentional engagement: content relevance and delivery accessibility. One participant stated:

“*Deciding whether to listen to a peer presentation mainly depends on two dimensions. One is content relevance… The other is delivery performance*.” (P03, interview).

When topics aligned with the current course unit or personal academic interests, participants reported greater willingness to invest attention. Presentations perceived as thematically peripheral were more readily deprioritized.

Delivery accessibility further shaped these judgments. Participants described losing attention when presentations were difficult to follow, especially because of dense slides or disorganized explanations. As one explained,

“*If a peer's PPT is full of text… or if the explanation is hesitant and logically disorganized, I find it hard to keep up, and my attention becomes distracted*” (P11, interview).

In such cases, attentional withdrawal reflected difficulty in sustained tracking rather than simple rejection of the topic.

These accounts suggest that audience attention depended not only on what was presented, but also on whether peer delivery supported continuous comprehension. Relevance and accessibility thus worked together as dimensions through which participants interpreted and regulated attentional engagement.

#### 4.3.2 Perceived payoff and attentional investment

Participants also described attention as a limited resource adjusted according to perceived payoff, cognitive effort, and competing academic demands. Several calibrated attention in relation to immediate learning priorities. One explained:

“*When I am in a high-intensity study period… I decide how much attention to invest based on the practical value of the presentation for my current learning*” (P15, interview).

When perceived value was low, participants reported reallocating attention to other tasks. As one noted,

“*I let myself get distracted for about ten minutes to organize materials for my own presentation and did not follow the peer presentation*” (P09, interview).

Conversely, when presentations offered concrete and transferable value, participants reported greater willingness to sustain attention. One recalled applying ideas from a peer presentation on film dialogue analysis to their own writing.

These accounts indicate that attention was interpreted and regulated through perceived instructional value: engagement was more likely to be maintained when benefits seemed to outweigh effort, and reduced when continued attention appeared to offer limited payoff.

### 4.4 Internal and external conditions shaping attentional vulnerability

#### 4.4.1 Internal conditions: vulnerability thresholds in the audience role

Participants frequently referred to internal states that constrained their perceived capacity to sustain attention, including fatigue, insufficient sleep, emotional strain, and reduced readiness. These conditions were not treated as direct causes of disengagement, but as factors that lowered attentional resilience once attention began to fluctuate.

Several participants linked attentional difficulty to fatigue and sleep deprivation. One stated:

“*I didn't sleep well the night before, so I felt very sleepy during class*.” (N42, written narrative)

Another noted:

“When I was very tired, my attention couldn't stay on the presentation for long” (N16, written narrative).

Emotional state was also reported as relevant. One participant explained,

“If my mood was not good that day, it was easier for me to lose focus” (N63, written narrative),

while another stated,

“*When I was feeling anxious or upset, I found it hard to concentrate on what my classmates were presenting*” (N21, written narrative.

Participants also referred to limited attentional readiness. As one explained,

“*Sometimes my mind was still thinking about other things, so I couldn't really get into listening*” (P05, interview).

In peer-presentation classrooms, where delivery quality and pacing varied across presenters, these internal vulnerabilities appeared to lower the threshold for disengagement and made participants more sensitive to presentational weaknesses.

#### 4.4.2 External conditions: presentational coherence and environmental disruptions

External conditions related to peer delivery and the classroom environment were also described as shaping attentional vulnerability. Participants reported difficulty sustaining attention when presentations lacked coherence, fluency, or audibility. One remarked:

“*The presenter kept stopping to look at the slides, and it was hard to follow their thinking*” (N12, written narrative).

Another noted:

“*The logic was not very clear, so after a while I didn't know what they were talking about*” (N47, written narrative).

Unclear articulation had a similar effect:

“*Sometimes I couldn't hear clearly what they were saying, and then I just stopped listening*” (N30, written narrative).

These accounts suggest that attentional withdrawal often followed difficulties in continuous tracking rather than immediate rejection of the topic itself. Environmental disruptions compounded these difficulties. Participants referred to outside noise, nearby peer conversations, and class timing as sources of interference. One explained:

“*There was noise outside the classroom, and people were talking nearby, which made it hard to concentrate*” (N55, written narrative).

Another noted:

“*When it was time for lunch, I was easily distracted thinking what should I have for lunch”* (N06, written narrative).

Because peer presentations temporarily assign instructional authority to students, external disruptions were not experienced simply as background interference. In participants' accounts, weak delivery, unclear organization, and environmental noise together signaled limited instructional support, increasing reliance on surface monitoring rather than sustained engagement.

### 4.5 Regulatory responses to attentional disruption

Participants described several responses to attentional disruption, reflecting their position as non-presenting peers who had to balance attentional effort, classroom norms, peer respect, and perceived instructional value.

#### 4.5.1 Self-initiated regulation: minimal-cost re-alignment

Some participants reported self-initiated regulation through low-effort strategies aimed at restoring minimal attentional alignment. These included bodily adjustment, visual anchoring, and surface-level note-taking. One participant described:

“*When my attention became distracted, I would sit up straight and try to observe my classmates or look at the PPT*” (P02, interview).

Another noted:

“*When I didn't want to listen, I would stare at the PPT and copy down key content to resist external interference*” (N39, written narrative).

Other participants described forcing attentive behavior even when internal engagement was weak. One explained:

“*I would force myself to look at the PPT or record keywords*” (N24, written narrative).

These accounts suggest that self-initiated regulation often maintained visible classroom participation and minimal tracking, rather than necessarily restoring deep comprehension.

#### 4.5.2 Externally triggered re-engagement: salience and accountability cues

Attention was also described as reactivated by external cues, especially presentation salience and classroom accountability. Participants reported returning attention when presentation format changed or when teacher intervention altered the attentional environment. One stated:

“*If interesting examples, videos, or interactive segments appeared in the PPT, my attention would usually come back*” (P14, interview).

Another emphasized teacher involvement:

“*When the teacher reminded us or guided the class, everyone's attention would be pulled back*” (N58, written narrative).

Environmental changes also supported re-engagement. One participant explained:

“*When the external noise disappeared, or when the presenter strengthened the visual and audio presentation, my attention would return*” (P10, interview).

These accounts indicate that re-engagement was often situationally induced. In peer-presentation contexts, teacher reminders were especially important because they temporarily restored formal accountability to an otherwise peer-led episode.

#### 4.5.3 Pragmatic disengagement: withdrawal of attention

When regulation failed or perceived instructional yield remained low, participants described partial withdrawal of attention. This was often framed not as impulsive distraction, but as pragmatic reallocation of cognitive resources. One participant explained:

“*Because the peer presentation was boring and the noise outside the classroom kept interfering, I let myself get distracted for about ten minutes and organized materials for my own presentation instead*” (P09, interview).

Another reported brief withdrawal followed by self-correction:

“*I would unconsciously scroll my phone for about one minute, and then force myself to pull my attention back to the class*” (P06, interview).

These accounts suggest that disengagement was sometimes narrated as a practical response when continued attention seemed to offer limited learning value or when regulatory costs exceeded expected benefits. Withdrawal therefore did not necessarily indicate indifference to classroom norms, but reflected participants' reported attempts to manage attention under weak instructional payoff and competing demands.

### 4.6 Evaluative outcomes and perceived cumulative effects in peer-presentation teaching

Participants frequently described forming post-presentation evaluations of the usefulness and legitimacy of peer presentations. These evaluations concerned whether peer presentations were meaningful learning experiences and whether they justified sustained attentional investment in later episodes.

#### 4.6.1 Value recognition paired with quality sensitivity

Many participants recognized the pedagogical value of peer presentations, especially for language learning, oral expression, and exposure to diverse topics. One stated:

“*I think classroom peer presentations are very valuable, because they allow presenters to practice oral English and expression, and also let us encounter diverse topics and cultures*” (P01, interview).

Another noted:

“*Peer presentations are a meaningful teaching format for both presenters and listeners*” (N66, written narrative).

However, this recognition was paired with strong sensitivity to presentation quality. Participants distinguished presentations that offered interpretation and insight from those that merely reproduced written materials. One explained:

“*If the presentation only lists the author's life and works without any analysis, it is just ‘moving text'*” (P13, interview).

Another similarly noted:

“*Most of the time, people are just reading the PPT content, and very little is based on their own understanding*” (N19, written narrative).

These accounts indicate that participants did not reject peer presentations as a pedagogical form. Rather, they evaluated instructional legitimacy according to whether presenters added explanatory or interpretive value beyond slide content. This pattern was coded as conditional acceptance: peer presentations were accepted as valuable when presenters added explanatory or interpretive value beyond slides.

#### 4.6.2 Normative and relational motivations in the audience role

Beyond learning value, some participants framed attentional behavior in normative and relational terms, emphasizing peer respect and classroom norms. One explained:

“*When classmates are presenting, I feel that I should listen*.” (N44, written narrative)

Another noted:

“*I would still try to listen, even if the content was not very interesting*” (N03, written narrative).

These accounts suggest that attentional regulation was not driven solely by cognitive or instrumental motives. Participants also described minimal attentiveness as social compliance and relational responsibility toward presenting peers. This helps explain why surface monitoring persisted even when comprehension declined: listeners could remain behaviorally attentive to uphold peer-respect norms, even when cognitive engagement weakened.

#### 4.6.3 Perceived cumulative effects and later expectations

Participants retrospectively linked repeated peer-presentation experiences to later expectations. Repeated exposure to low-quality presentations was described as weakening perceived instructional value. One participant reflected:

“*When many presentations are squeezed together, there is not enough energy to do each one well, and in reality, about half of the students do not really listen*” (P04, interview).

Another noted:

“*In the end, people only really understand the content of their own presentation, and do not know much about others' content even after it is finished*” (N71, written narrative).

At the same time, participants indicated that high-quality presentations could reinforce perceived legitimacy. One observed:

“*When the presenter speaks fluently and the PPT is well-designed and interesting, the whole class becomes very focused*.” (N25, written narrative).

These accounts suggest a perceived cumulative pattern rather than a demonstrated longitudinal effect. Participants described past presentation experiences as informing their expectations, attentional thresholds, and willingness to invest effort in subsequent presentations.

### 4.7 A situated process model of perceived audience attention

**Figure 1:**
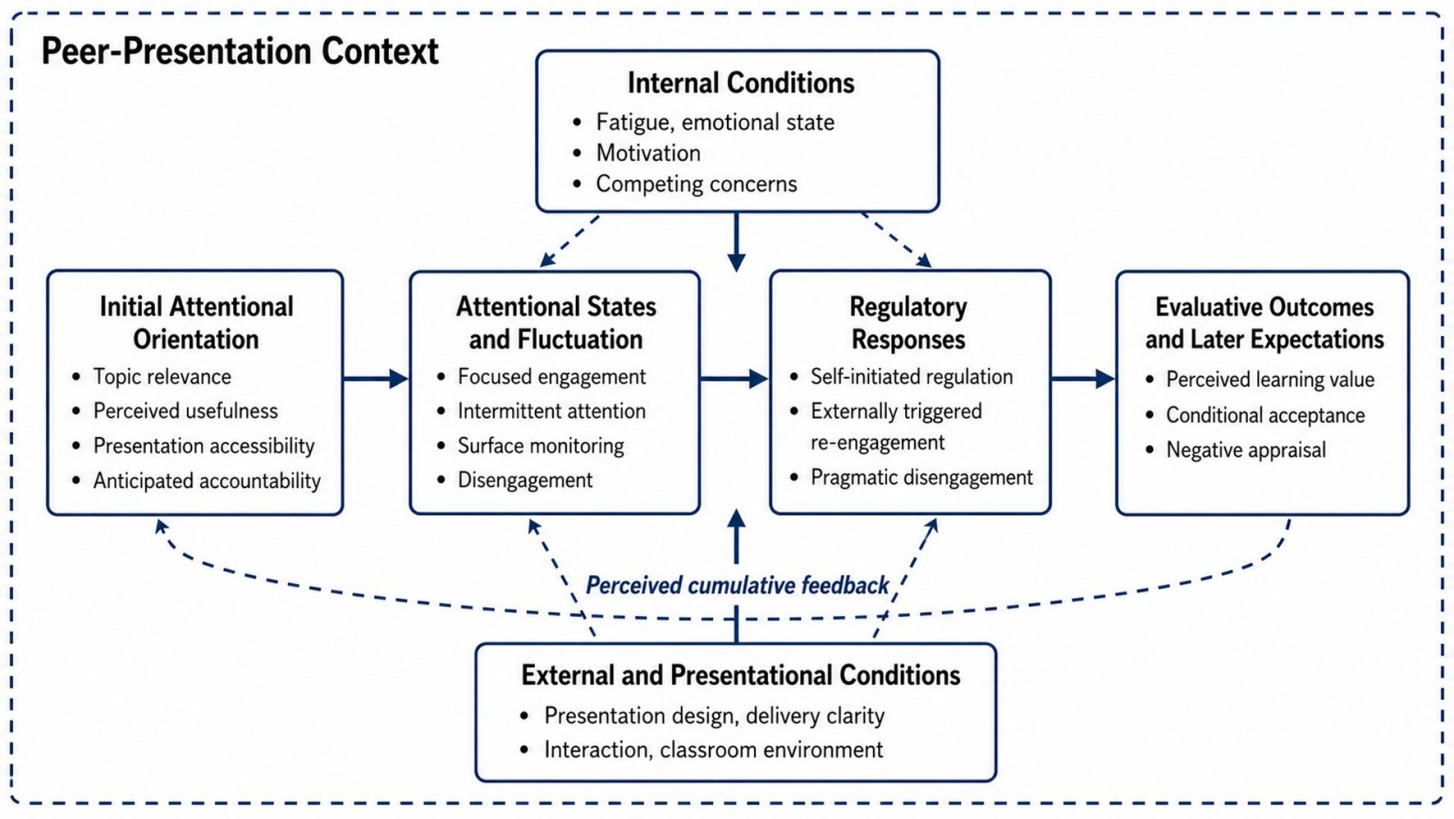
A situated process model of perceived audience attention during classroom peer presentations. Solid arrows indicate a commonly reconstructed process sequence; dashed arrows indicate conditional influences and perceived feedback rather than fixed causal effects.

Integrating the findings, a situated process model of perceived audience attention during classroom peer presentations was developed (see Figure 1). The model synthesizes initial attentional orientation, attentional fluctuation, regulatory responses, and evaluative outcomes as reconstructed across participants' accounts. It is grounded in peer-presentation contexts, where instructional authority is temporarily redistributed and audience members navigate learning goals, peer-respect norms, and limited accountability.

As shown in Figure 1, attention was initially oriented through perceived topic relevance, usefulness, presentation accessibility, and anticipated accountability. It then fluctuated across focused engagement, intermittent attention, surface monitoring, and disengagement. Disruption prompted self-initiated regulation, externally triggered re-engagement, or pragmatic disengagement when continued listening seemed to offer limited payoff. Internal conditions, such as fatigue, emotional state, motivation, and competing concerns, shaped participants' perceived capacity to sustain attention, while external and presentational conditions shaped whether peer-delivered input was experienced as accessible and worthwhile.

The arrows are grounded in recurring patterns, but they do not imply that every participant followed the same sequence. Some cases followed the full process: initial interest weakened when explanation became difficult to follow, participants used the PPT as a visual anchor, and Q&A later restored relevance. Other cases followed the sequence only partly, moving from weak initial relevance directly to surface monitoring. A third pattern involved resistance to disengagement, as some participants continued looking at slides or recording keywords because of peer-respect norms despite limited comprehension.

The feedback loop should therefore be understood as a perceived cumulative pattern rather than a demonstrated longitudinal effect. Participants retrospectively linked repeated exposure to low- or high-quality presentations with later expectations about whether peer presentations were worth sustained attention. The model thus shows how attentional fluctuation, regulation, and evaluation are configured together in peer-led contexts marked by provisional authority, variable presentation quality, peer-relational norms, and limited audience accountability.

## 5 Discussion

This study conceptualized audience attention as a situated classroom phenomenon embedded in peer presentations. Rather than proposing a theory of attention in isolation from existing research, the findings position the model as a situated extension of classroom attention, mind-wandering, self-regulation, and engagement frameworks. Unlike teacher-led instruction, peer presentations redistribute instructional authority, positioning student presenters as provisional knowledge providers and non-presenting peers as audience members whose attention is less institutionally enforced. This role configuration means that attention cannot be assumed as a default classroom behavior, but is perceived and narrated as actively negotiated. The study extends prior attention research, which has largely examined teacher-fronted settings and conceptualized attentional lapses primarily as cognitive failures or mind-wandering episodes (Risko et al., 2012; Smallwood and Schooler, 2015). Here, disengagement often reflected situated judgments about the legitimacy, relevance, and accessibility of peer-delivered knowledge.

The findings align with research on attention and mind wandering as dynamic processes (Risko et al., 2012; Yang et al., 2025), but show that fluctuation takes on particular significance in peer-presentation contexts. Whereas, L2 classroom studies trace developmental trajectories of mind wandering across lectures (Yang et al., 2025), the present study shows that fluctuation in peer presentations is also shaped by uneven presentation quality, brief but repeated presentation segments, limited interaction, and weak audience accountability. Surface monitoring and partial engagement can therefore be understood as adaptive forms of classroom presence rather than simple deficits.

The findings also highlight the evaluative nature of audience attention. Participants described attention in relation to topic relevance, comprehensibility, and perceived instructional payoff. While peer instruction supports cognitive engagement under structured conditions (King, 2002; Tullis and Goldstone, 2020), peer presentations involve epistemic ambiguity: presenters are simultaneously learners and temporary knowledge authorities. This ambiguity may lower the threshold for withdrawal when peer-delivered input fails to meet expectations. The study therefore adds a social–epistemic dimension to attention research by showing how perceived authority, competence, and instructional legitimacy shape attentional investment.

Participants further described self-initiated regulation, externally triggered re-engagement, and pragmatic disengagement. These responses reflect the audience role: unlike presenters, audience members are not accountable for delivery, so their regulation often prioritizes minimal compliance, peer respect, and cognitive efficiency. This finding resonates with engagement research distinguishing surface participation from deeper cognitive involvement (Fredricks et al., 2004; Skinner and Pitzer, 2012), while suggesting that disengagement may be narrated as a practical response to low instructional yield. Teacher reminders may temporarily restore attention, but are unlikely to sustain engagement without meaningful instructional support.

Consistent with research on attentional vulnerability (D'Mello, 2019; Rajeswari et al., 2025), internal states such as fatigue and reduced motivation were described as heightening susceptibility to attentional breakdown. These internal conditions interacted with external presentation features: weak support amplified vulnerability, whereas clear organization and interaction partly compensated for it. This extends mind-wandering research by showing how instructional design mediates attentional regulation where instructional authority is decentralized.

These findings suggest that uneven attention is not simply a matter of student motivation but also an instructional design issue. Instructors can support audience attention by clarifying presentation requirements, assigning concrete listening tasks, and offering light pre-presentation feedback. Presenter preparation should address low audibility, unclear slides, dense text, and weak transitions. Brief interaction points, teacher framing, concise feedback, and careful scheduling can further strengthen accessibility, accountability, and perceived payoff.

## 6 Conclusion

This study examined audience attention in classroom peer presentations through a grounded theory-informed qualitative approach, focusing on how non-presenting peers perceived, reconstructed, regulated, and evaluated attention. The findings show audience attention as a dynamic, evaluative, and recursive process shaped by individual states, presentational conditions, and classroom norms.

Across participants' accounts, attention fluctuated between focused engagement, intermittent attention, surface monitoring, and disengagement. These shifts reflected judgments about the relevance, accessibility, and legitimacy of peer-delivered content. Disengagement was often narrated not as simple inattentiveness, but as a pragmatic response to low instructional value or excessive cognitive cost.

The proposed model conceptualizes perceived audience attention as a cyclical system involving orientation, fluctuation, regulatory responses, and evaluative feedback. It is not intended to replace existing theories of attention, engagement, mind wandering, or self-regulation; rather, it shows how broader attentional processes take on specific meanings in peer-led contexts, where authority and accountability are redistributed. Participants also retrospectively linked repeated presentation experiences to later expectations, suggesting a perceived cumulative pattern rather than a demonstrated longitudinal effect.

Several limitations should be acknowledged. The study was conducted in undergraduate courses at a single institution, so the findings may reflect specific cultural, institutional, and classroom norms. It relied mainly on interviews and written narratives, although these were collected immediately after class sessions. Observation-informed sampling may also have privileged visible attentional changes. Future research may examine the model across different institutions and disciplines and combine qualitative accounts with observational or real-time measures.

Overall, this study contributes to classroom attention and peer learning research by foregrounding audience attention as situated and role-dependent. By shifting focus from presenter performance to audience experience, it explains why peer presentations often yield uneven engagement and suggests that instructors design peer presentations with clearer audience tasks, stronger presenter preparation, interaction points, and supportive scheduling.

## Data Availability

The original contributions presented in the study are included in the article/supplementary material, further inquiries can be directed to the corresponding author.
